# Interrater Agreement on National Institutes of Health Stroke Scale Between Paramedics and Stroke Physicians: Validation Study for the Digital Training Model in the Paramedic Norwegian Acute Stroke Prehospital Project

**DOI:** 10.2196/39444

**Published:** 2022-08-11

**Authors:** Mona Guterud, Helge Fagerheim Bugge, Jo Røislien, Karianne Larsen, Erik Eriksen, Svein Håkon Ingebretsen, Martin Lerstang Mikkelsen, Jo Kramer-Johansen, Kristi G Bache, Else Charlotte Sandset, Maren Ranhoff Hov

**Affiliations:** 1 Department of Research Norwegian Air Ambulance Foundation Oslo Norway; 2 Division of Prehospital Services Oslo University Hospital Oslo Norway; 3 Institute of Clinical Medicine University of Oslo Oslo Norway; 4 Department of Neurology Stroke Unit Oslo University Hospital Oslo Norway; 5 Institute of Basic Medical Sciences University of Oslo Oslo Norway; 6 Research and Dissemination Østfold University College Halden Norway; 7 Department of Health Science Oslo Metropolitan University Oslo Norway

**Keywords:** paramedic, stroke, ambulance, National Institutes of Health Stroke Scale, NIHSS, training, digital, interrater, agreement, Norway, acute, treatment, hospital, time, communication, ambulance, mobile application, clinical trial, physician, digital, simulation

## Abstract

**Background:**

Time spent in the prehospital phase of acute stroke care is multifactorial and has an effect on the possibilities for acute treatment. Communication between paramedics and the in-hospital stroke team directly affects time to treatment. A mutual stroke scale such as the National Institutes of Health Stroke Scale (NIHSS) may improve communication quality. The Paramedic Norwegian Acute Stroke Prehospital Project (ParaNASPP) was a stepped-wedge, randomized trial of stroke screening using NIHSS in the ambulance where the intervention was training paramedics in stroke and the NIHSS, with the use of NIHSS made into a mobile app to guide the examination and facilitate communication with the in-hospital stroke team.

**Objective:**

The aim of this study was to validate the digital training model from the ParaNASPP clinical trial.

**Methods:**

In total, 24 paramedics were recruited from Oslo University Hospital in Norway to complete the ParaNASPP training model; 20 exclusive videos with predefined NIHSS scores were recorded; and 4 stroke physicians from Oslo University Hospital were included for reference. Bland-Altman plots with 95% limits of agreement (LoA) were calculated—first comparing paramedics and stroke physicians to the predefined scores and then with each other. The predefined LoA were set to 3 points. To align with clinical practice, NIHSS scores were also dichotomized into 2 categories: from 0-5 (minor stroke) or ≥6 (moderate and major stroke), and agreement was calculated using Cohen *κ*.

**Results:**

The videos (n=20) had a median (range) NIHSS score of 7 (0-31). The paramedics’ scores were slightly higher than the predefined scores with a mean difference of –0.38 and the LoA ranging from –4.04 to 3.29. The paramedics scored higher than the stroke physicians with a mean difference of –0.39, with the LoA ranging from –4.58 to 3.80. When the NIHSS scores were dichotomized, Cohen *κ* was 0.89 between the predefined scores and paramedics, 0.92 between the predefined scores and stroke physicians, and 0.81 between the paramedics and stroke physicians, all indicating very good agreement.

**Conclusions:**

The paramedics scored higher than both the predefined scores and stroke physicians’ scores, hence the predefined LoA were not met. However, the width of the LoA was smaller than seen when experienced neurologists are compared. When the NIHSS scores were dichotomized, the paramedics achieved very good agreement with both the predefined scores and stroke physicians’ scores. This study demonstrates the possibilities for the transfer of clinical competence in digital simulation training.

## Introduction

The correct and timely triage of patients with acute stroke to the right level of care is largely based on the prehospital assessment [1,2]. Prehospital stroke symptom identification and the prenotification of in-hospital stroke teams are known to affect time to acute treatment [3,4]. Prenotification communication with the receiving facility is important as it prepares the stroke team on the patient’s condition and secures the efficient in-hospital reception of the patient [2,4]. The National Institutes of Health Stroke Scale (NIHSS; Multimedia Appendix 1) is the most frequently used stroke scale by stroke physicians and stroke nurses today [5]. The NIHSS has been considered too complex and time-consuming and, therefore, less suited for prehospital use [6,7], and consequently, most prehospital scales are the modified and shortened versions of the NIHSS [8,9]. Fair agreement has been found when comparing the NIHSS scores achieved by neurologists and nonneurologists [10-12], but little is known on how the full-scale NIHSS when performed by paramedics compare to stroke physicians’ scores. Traditional simulation training is to a large degree based on physical attendance and, thus, is both time- and resource-consuming. Alternative solutions for training medical personnel, including video-based training, have been investigated [13,14] and proven to be reliable in NIHSS training and certification [15,16]. Video-based training supplemented with electronic learning (e-learning) has shown better performance in NIHSS scoring [17]. For the Paramedic Norwegian Acute Stroke Prehospital Project (ParaNASPP)—a stepped-wedge, randomized trial of stroke screening using NIHSS in the ambulance—we developed a complete digital training model for paramedics [18]. An e-learning program was combined with unique videos for scoring NIHSS in the (native) Norwegian language.

The aim of this study was to validate the training model in the ParaNASPP clinical trial.

## Methods

### Study Setting

In the ParaNASPP clinical trial [18], paramedics in Oslo, Norway, were trained in the full-scale NIHSS as the intervention. The participant enrollment period was from June 3, 2019, to July 1, 2021. The intervention included a structured learning program, a mobile app for NIHSS scoring, and the transfer of data from paramedics to the on-call stroke team physician. In October 2018, we tested the intervention for feasibility and identified the needed adjustments in the e-learning and simulation training before the start of the trial. To validate the training model, we decided to test the interrater agreement between paramedics and stroke physicians, and we planned for a pilot study. Due to the COVID-19 pandemic, a need for digital training emerged. For practical reasons, we decided to test the interrater agreement after digital simulation training.

The validation study took place in Oslo, Norway, in December 2020. Due to pragmatic and organizational reasons, we invited all (N=83) ambulance personnel employed at 3 geographically dispersed ambulance stations in the Prehospital Division of Oslo University Hospital to participate. To become an ambulance personnel in Norway, there is emergency medical technician training from upper secondary school. Paramedic training may be accomplished for emergency medical technicians and nurses with additional courses, and in recent years, a unique bachelor’s degree for paramedics has been developed as a higher education. To reflect the diversity in the ParaNASPP clinical trial study setting [18], we needed participants from this spectrum. For simplicity, we refer to the group as paramedics. Based on current protocol in the ambulance service, we expected the paramedics to have no or little formal competence or experience with the NIHSS. For comparison, selected stroke physicians that reflected the variations in the on-call team at the Stroke Unit of the Department of Neurology at Oslo University Hospital were also asked to participate.

Data that were collected from the participants included the number of years of experience in their respective services, level of education, and current status on the international certification in NIHSS [16]. Written consent was obtained from all participants.

### Practical Implementation

All enrolled paramedics completed a structured e-learning program in stroke assessment prior to a live, digital simulation training on the Teams chat-based collaboration platform (version 4.7.15.0; Microsoft). The digital simulation training lasted 4 hours. A stroke physician tutored the sessions, where the aim was to build an understanding of the assessment of neurological findings, the concept of the NIHSS, and the practical use of a mobile iOS app (the ParaNASPP app; Multimedia Appendix 2). This is a specially developed app where each item from the NIHSS is displayed in pictograms, explanatory text is presented in a fixed sequence, and a total score is automatically calculated. A separate validation study of the ParaNASPP app has been published [19]. All items in the NIHSS were demonstrated and simulated. Simulation cases in the live stream were unique and distinct from the forthcoming, predetermined cases to test the interrater agreement. The participants could ask questions, and they received immediate feedback and guidance from the instructors and stroke physician. Immediately after the live stream of the digital simulation training, the paramedics accessed the test material for the study.

In all, 20 exclusive videos (see an example in Multimedia Appendix 3) with the role-playing of the neurological symptoms of a possible acute stroke were developed and used for testing interrater agreement. To achieve a trustworthy acting of neurological findings, a stroke physician performed as the patient in all videos, and a paramedic trained in the ParaNASPP model [18] performed the NIHSS examination. The manuscripts for the videos were prepared in cooperation with stroke physicians who were not involved in this study. The video manuscripts represented the predefined NIHSS scores with a median (range) of 7 (0-31). The videos were intended to comprise the different items of the NIHSS to varying degrees; however, the cases of neurological findings not captured in the NIHSS were also acted out, such as dizziness and dysmetria. The distribution aimed to reflect a real population with stroke [20] and was similar to comparable studies [11,15]. The videos had a mean (SD) duration of 2 minutes and 58 (23) seconds. The videos could be paused and rewound if warranted by the participants. When the paramedics scored the last NIHSS item in the app, a total score was transferred to the database, and this finalized the scoring opportunity for that video.

The paramedics’ NIHSS scores were compared to the predefined scores for each video. As this underlying predefinition is not available in clinical practice, the paramedics’ scores were also compared to the scores achieved by stroke physicians scoring the same videos.

All paramedics’ NIHSS scores were digitally entered in the ParaNASPP app. The time spent on NIHSS registration was recorded by start time (new registration) and end time (data submitted) and directly transferred to the database. The stroke physicians scored according to their daily practice on the original NIHSS paper form, independently from each other and the paramedics. The stroke physicians were responsible for documenting their own time stamps for each video. The time spent on scoring the NIHSS for each video was reported in whole minutes.

### Statistical Analysis

We presented continuous data as mean (SD) for symmetric data and median (range) for skewed data and data with outliers.

The NIHSS is a continuous scale, and Bland and Altman’s [21] approach for method comparison was applied to assess the interrater agreement. The limits of agreement (LoA) were estimated based on the observed differences between measurement methods, representing the actual variation in the data [22]. These LoA were then compared to the acceptable variation, here set to 3 points on the NIHSS based on a clinical evaluation and the same a priori threshold in a comparable study [14]. Bland and Altman’s [21] original method was applied when comparing the NIHSS scores between the paramedics or stroke physicians and the predefined scores in the videos. When assessing the interrater agreement between the paramedics and stroke physicians, a mixed models version of method comparison was applied [23], adjusting for the internal correlation structure in the data resulting from the 24 paramedics and 4 stroke physicians all evaluating the same 20 videos.

In clinical practice, a distinction in treatment regimens is often made for high versus low NIHSS scores [24,25], and thus, in a secondary analysis, the interrater agreement for dichotomized NIHSS values were explored. The continuous NIHSS scores were dichotomized into a low-score category, from 0-5 (minor stroke), and a high-score category, ≥6 (moderate and major stroke). Cohen *κ* was used to calculate the agreement of the dichotomized data: first, between the paramedics or stroke physicians and the predefined scores and second, between the paramedics and stroke physicians. Note that currently, no version of the mixed models of Cohen *κ* exists, and the traditional Cohen *κ* used will likely underestimate the uncertainty in the Cohen *κ* estimate.

In the literature, *κ*≤0.2 is taken to represent poor agreement, 0.21-0.40 as fair agreement, 0.41-0.60 as moderate agreement, 0.61-0.80 as good agreement, and 0.81-1.0 as very good agreement [26].

Statistical analyses were performed with Stata statistical software (version 16.1; StataCorp) [27] and R statistical software (version 4.0.3; R Foundation for Statistical Computing) [28].

### Ethical Considerations

The local data protection office at Oslo University Hospital approved of the handling of the data from the volunteers and consenting paramedics and stroke physicians employed at Oslo University Hospital (approval 19/00667). No institutional review board approval was sought since no actual patients were involved in this study, as outlined by Norwegian guidelines.

## Results

This study enrolled all (N=24) paramedics that volunteered and recruited 4 volunteer stroke physicians. The characteristics of the participants are described in Table 1.

Time spent on evaluating the videos contained 2 extreme values (196 minutes and 5768 minutes), likely a result of starting a video, pausing, and completing it at a later time point. These outliers were therefore excluded from the analysis.

**Figure 1:**
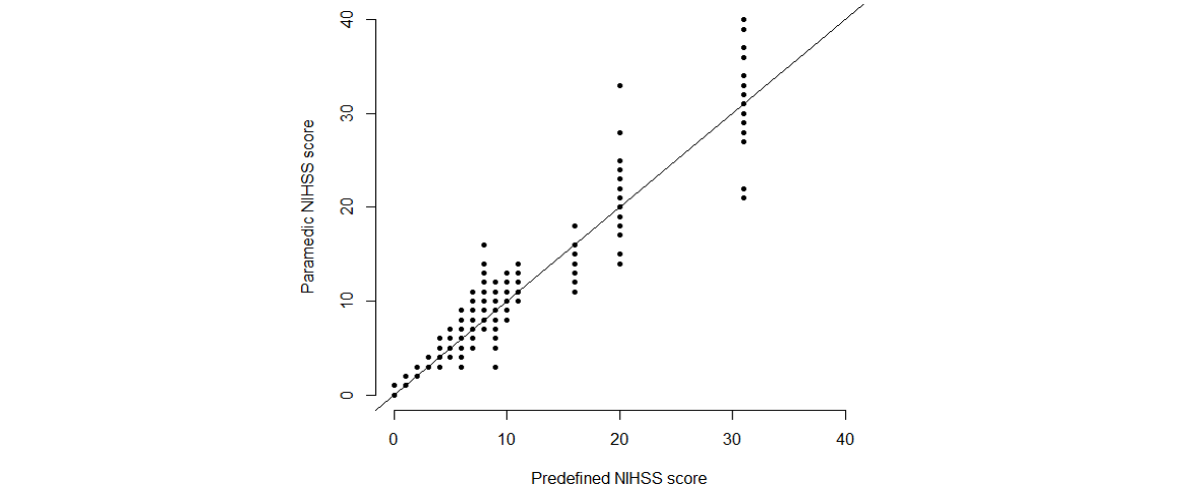
NIHSS scores for the paramedics against the predefined NIHSS scores (raw data). NIHSS: National Institutes of Health Stroke Scale.

**Figure 2:**
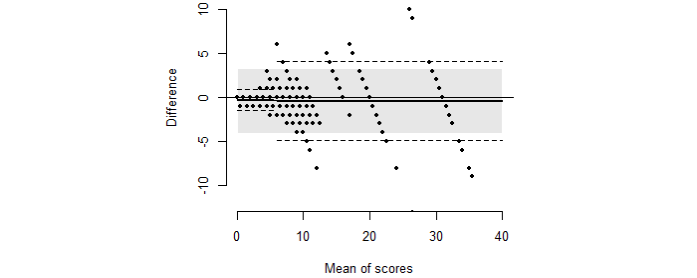
NIHSS scores for the paramedics against the predefined NIHSS scores with the corresponding Bland-Altman plot displaying pairwise differences plotted against pairwise means. The limits of agreement are superimposed, calculated both for the total data sample (shaded) and for the from 0-5 versus ≥6 categories separately (dashed line). NIHSS: National Institutes of Health Stroke Scale.

Comparing the paramedics’ score to the predefined scores in the videos resulted in 480 unique NIHSS assessments. Similarly, the stroke physicians enrolled in the study’s evaluation of the 20 videos resulted in 80 unique NIHSS scores. The paramedics’ scores were on average somewhat higher than the predefined scores (Figure 1), with a mean difference of –0.38 and the LoA ranging from –4.04 to 3.29 between the paramedics’ scores and the predefined scores (Figure 2). The paramedics scored higher than the stroke physicians, with a mean difference of –0.39 and the LoA ranging from –4.58 to 3.80. The agreements between the paramedics’ scores with the predefined scores and stroke physicians’ scores were both outside the a priori defined acceptable limit of 3.

**Figure 3:**
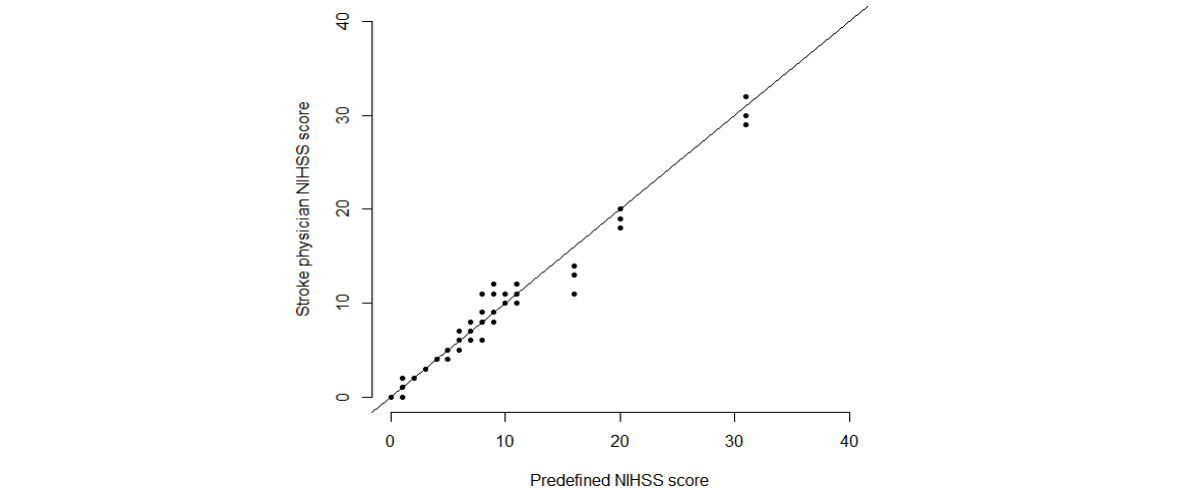
NIHSS scores for the stroke physicians against the predefined NIHSS scores (raw data). NIHSS: National Institutes of Health Stroke Scale.

**Figure 4:**
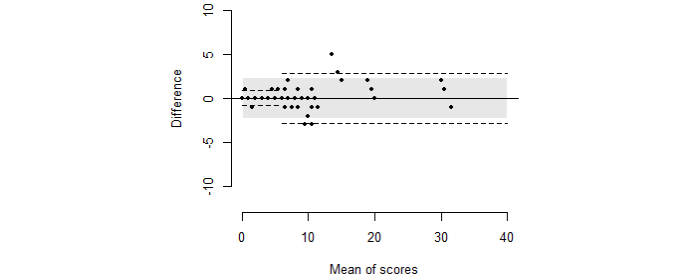
NIHSS scores for the stroke physicians against the predefined NIHSS scores with the corresponding Bland-Altman plot displaying pairwise differences plotted against pairwise means. The limits of agreement are superimposed, calculated both for the total data sample (shaded) and for the from 0-5 versus ≥6 categories separately (dashed line). NIHSS: National Institutes of Health Stroke Scale.

The stroke physicians were in agreement with the predefined scores (Figure 3), and the LoA ranged from –2.31 to 2.34 with a mean difference of 0.01, which were well within the limit of 3 (Figure 4).

Differences between the paramedics’ scores and the predefined scores in the videos were considerably smaller for lower NIHSS scores. Calculating the LoA for the 2 clinically different regions, we found the LoA to be from –1.42 to 0.88 for NIHSS scores from 0-5 and from –4.90 to 4.03 for NIHSS scores ≥6 (Figure 2).

The paramedics’ ability to score patients in the from 0-5 or ≥6 categories showed a Cohen *κ* of 0.89 as compared to the predefined scores, representing very good agreement. For predefined scores from 0-5, 14 (8.3%) out of 168 paramedics’ scores were overestimated, putting patients in the high-score category. For predefined scores ≥6, the paramedics’ scores were underestimated in 9 (2.9%) out of 312 videos.

**Table 1:** Description of the participants.

Characteristic		Paramedics (N=24)	Stroke physicians (N=4)
Experience (years), median (range)		4 (1-45)	11 (8-14)
Time in a stroke unit (years), median (range)		—^a^	7 (2-10)
**Level of education, n (%)**			
	EMT^b^	8 (33)	—
	Trained paramedics	14 (58)	—
	Apprentice EMT	1 (4)	—
	Other	1 (4)	—
	Specialist in neurology	—	3 (75)
	Specialist in geriatric medicine	—	1 (25)
Certification in NIHSS^c^, n (%)		5 (21)	4 (100)
Time spent on each case (minutes), median (range)		3 (2-15)	3 (2-4)

^a^Not applicable.

^b^EMT: emergency medical technician.

^c^NIHSS: National Institutes of Health Stroke Scale.

Interrater agreement between the stroke physicians’ scores and the predefined scores for the 2 categories was *κ*=0.92, representing very good agreement. When the predefined scores were from 0-5, the physicians were in complete agreement with predefined scores in 28 (100%) out of 28 videos, and when the predefined scores were ≥6, the stroke physicians’ scores were underestimated in 3 (6%) out of 52 videos.

With 20 predefined scores, 24 paramedics, and 4 stroke physicians, we had 1920 paired NIHSS score comparisons which gave an unadjusted Cohen *κ* of 0.81 and very good agreement in the direct comparison between the paramedics and stroke physicians. The paramedics scored the simulated patients to be in the ≥6 category while the stroke physicians scored in the from 0-5 category in 128 (17.2%) out of 744 comparisons. The opposite occurred in 36 (3.1%) out of 1176 comparisons.

## Discussion

### Principal Findings

Our findings indicate that paramedics can achieve very good agreement with stroke physicians when tested after a digital training program for NIHSS in the ParaNASPP model.

The paramedics scored higher than both the predefined scores and the stroke physicians’ scores when we looked at the scale from 0 to 42 points. Compared to the predefined scores, the paramedics were well within the LoA of 3 in the range of NIHSS scores from 0-5; however, the variation increased with higher scores (≥6). Higher NIHSS scores indicate more complex neurological deficits [5] and have been associated with greater scoring variance in other settings, and a difference of 4 points is not uncommon in video scoring [29]. Nevertheless, we had predefined an acceptable difference in scores of 3 points between raters. This is the same predefined limit used in a study to compare the NIHSS scores of remote and bedside vascular neurologist [14].

In this study, the participants were a heterogenous group, but it was important to test the training model on a group similar to that in the ParaNASPP clinical trial [18]. However, the width of 8.38 on the LoA for the paramedics’ and stroke physicians’ scores found in our study is smaller than seen when compared to experienced neurologists who achieved a width of 10.05 on the LoA [14]. A grading table for acceptable LoA has been developed, placing the results from our study as Grade A [30]. Based on this, we accept the LoA in our study in spite of not achieving the predefined limit.

The NIHSS scale ranges from 0 to 42 points where higher scores indicate more severe strokes [5] and more complex scoring, but a single number on a scale, or a category when it is applied, is never decisive of treatment. However, prehospital triage decisions are to some extent dependent on this scoring. We decided on a cutoff of 6 points for dichotomizing the scale to be in accordance with a cutoff commonly used [24,31,32]. In a clinical setting, there is an acceptance for overtriage to ensure the identification of patients eligible for acute treatment [7]. An overestimation of a NIHSS score or category from paramedics is for that reason less problematic than an underestimation, which in our study also was lower than seen before [6].

When dichotomized to from 0-5 and ≥6 categories, interrater agreement was very good between the paramedics’ scores and the predefined scores. Although a generalization of Bland and Altman’s [21] approach for the method comparison of continuous measurements is more than a decade old, when adjusting for replicate measurements and multiple raters, no readily available generalization for Cohen *κ* exists. However, a crude estimate for comparing categorized NIHSS scores between the paramedics and stroke physicians, combining all value pairs in the same cross table, gave an unadjusted Cohen *κ* that indicated very good agreement. When not in agreement, the tendency was shifted toward higher NIHSS scores representing the less problematic overtriage from the paramedics.

The duration of evaluating each case referred to the scoring of the simulated symptoms on the videos and does not necessarily reflect the time spent on performing the actual assessment. The stroke physicians scored the videos according to their daily practice with a self-report on case duration, whereas the paramedics were provided with an unfamiliar stroke scale and a new scoring tool that automatically registered case duration. We expected the paramedics to spend more time on scoring the videos based on the novelty, but the time spent did not differ much between the paramedics and stroke physicians. This finding may indicate an instant effect of our training model for the paramedics—an effect that may be sustained [33]. However, the scoring was based on the acting of neurological symptoms that were straight forward and not influenced by confounders seen in a real-world setting. The time spent on patient evaluation may increase for paramedics in a more complex clinical context.

The training of paramedics in acute stroke assessment can easily be converted to a digital format instead of on-site training [34]. Digital solutions have been suggested as an alternative to face-to-face interactions in simulation training [13], and significant correlation between digital solutions and positive learning outcomes have already been established [17,35]. This knowledge is important when planning for the implementation of new procedures and tools for paramedics. However, the supervision part of digital training is important [36]. A chat function makes the instructors available and provides a great opportunity for participants to interact despite their remote participation.

Recent publications demonstrate reasonable agreement between prehospital and in-hospital NIHSS scores, in both the modified and full-scale versions [30,34]. Importantly, paramedics preferred a hospital-based stroke scale to improve communication with stroke physicians [34]. The development of stroke triage systems has not focused on the standardization of clinical evaluation and communication between paramedics and the on-call stroke physician. Communication quality between paramedics and the on-call stroke team physician directly influences prehospital on-scene time and is a key component in prenotification and triage [37]. Introducing a common clinical language through training paramedics may facilitate this communication [15,37]. We believe that a solid training program is the key to standardizing clinical assessment in acute stroke care and that the reliable use of the NIHSS is related to how paramedics are trained rather than the profession itself. A compatible stroke scale will improve prehospital to in-hospital communication and the quality of the prenotification but also holds the potential to improve triage, optimize in-hospital reception, and reduce time to treatment. The ParaNASPP clinical trial [18] aims to investigate this.

### Limitations

This study was delayed due to organizational issues during the COVID-19 pandemic, and time limits and the pandemic affected our possibilities to engage a larger group.

We decided to use a stroke physician to perform as the patient in the videos to achieve a trustworthy acting of neurological findings. We realize that this is also a limitation as neurological findings in a real-world setting may be influenced by comorbidities, complicating the patient assessment. The results on the interrater agreement achieved in this study may therefore not be directly transferrable to a clinical setting.

The study was performed using a convenience sample, and an a priori power analysis was not performed. The low number of assessments between neurologists and video or paramedics might thus make the Bland-Altman analysis underpowered, with the accompanying increased uncertainty in the LoA estimates.

Only the total NIHSS score, and not the specific NIHSS score for each of the 11 score items, were available for analysis for the paramedics, and as a consequence, we were not able to identify if there were specific items that affected the agreement.

Failing to stay inside the predefined LoA of 3 is fundamentally different depending on if we are evaluating the lower or higher range of the NIHSS score. For future studies, it would be interesting to investigate if a shifting LoA acceptability and different cutoffs for dichotomizing the scale would alter the interrater agreement.

There were 5 paramedics who reported that they had an international certification in NIHSS. The NIHSS was not a part of standard protocol for paramedics, and the rather high proportion of paramedics with extracurricular knowledge may have contributed to a selection bias, since paramedics already interested in the topic were more likely to respond to the advertisement.

### Conclusion

The paramedics scored higher than both the predefined scores and the stroke physicians’ scores, hence the predefined LoA were not met. However, the width of LoA was smaller than seen when experienced neurologists are compared. When the NIHSS scores were dichotomized, the paramedics achieved very good agreement with both the predefined scores and the stroke physicians’ scores. This study demonstrates possibilities for the transfer of clinical competence in digital simulation training. It may facilitate training and implementation in greater scales in different prehospital services and improve the efficacy of training in the future.

## Appendix

Multimedia Appendix 1 National Institutes of Health Stroke Scale, English and Norwegian versions.

Multimedia Appendix 2 The Paramedic Norwegian Acute Stroke Prehospital Project (ParaNASPP) app with pictograms.

Multimedia Appendix 3 Example video.

## Abbreviations

e-Learning: electronic learning
LoA: limits of agreement
NIHSS: National Institutes of Health Stroke Scale
ParaNASPP: Paramedic Norwegian Acute Stroke Prehospital Project
