# Supplementary material for: Interrater Agreement on National Institutes of Health Stroke Scale Between Paramedics and Stroke Physicians: Validation Study for the Digital Training Model in the Paramedic Norwegian Acute Stroke Prehospital Project
Source: JMIR Neurotechnol. 2022 Aug 11;1(1):e39444. doi: 10.2196/39444 (PMC12671290; doi:10.2196/39444)
Supplement: Multimedia Appendix 1 [file neuro_v1i1e39444_app1.pdf]

National Institutes of Health Stroke Scale, English and Norwegian versions.

**NIH Stroke Scale (NIHSS)**	**Ambulance**	**Admission**	**2hrs**	**Day1**	**Discharge**
Use the first response as measurement of function<br>Do not instruct the patient (unless specified in the text)	Date				
Some parameters are only scored if present (i.e. limb ataxia)<br>Register how the patient performs, not what you think the patient can do	Time				
1a. Level of consciousness<br>0=Alert<br>1=not alert, but arousable by minor stimulation<br>2=not alert, requires repeated stimulation to attend<br>3=reflex motor or autonomic effects or unresponsive					
1b. Orientation (month + age)<br>0=answers bot questions correctly<br>1=answers one question correctly<br>2=answers neither question correctly					
1c. Response to command (close eyes, make fist)<br>0=performs both tasks correctly<br>1=performs one task correctly<br>2=performs neither task correctly					
2. Best gaze (eyes open, patient follows examiner's finger)<br>0=normal<br>1=partial gaze palsy, gaze is abnormal in one or both eyes.<br>2=forced deviation or total gaze paresis					
3. Visual fields<br>0=no visual loss<br>1=partial hemianopia<br>2=complete hemianopia<br>3=bilateral hemianopia (blind)					
4. Facial paresis (show teeth, raise eyebrows, squeeze eyes shut)<br>0=normal symmetrical movements<br>1=flattened nasolabial fold, asymmetry on smiling<br>2=total or near-total paralysis of lower face<br>3=complete paralysis of one or both sides (or in comatose patients)					
5. Motor arm (arm 45 degrees for 10 seconds)<br>0=no drift, arm holds 45 degrees for 10 seconds (also if not testable)<br>1=drift, arm holds 45 degrees, but drifts down	Left				
2=some effort against gravity, drifts to bed<br>3=no effort against gravity, limb falls<br>4=no movement	Right				
6. Motor leg (leg 30 degrees for 5 seconds)<br>0=no drift, leg holds 30 degrees for 5 seconds (also if not testable)<br>1=drift, leg holds 30 degrees, but drifts down	Left				
2=some effort against gravity, drifts to bed<br>3=no effort against gravity, limb falls<br>4=no movement	Right				
7. Limb ataxia (finger-nose test/heel-knee test)<br>0=absent (normal) (also if not testable or in comatose patients)<br>1=present in arm or leg<br>2=present in arm and leg					
8. Sensory (skin sensation)<br>0=normal, no sensory loss<br>1=mild to moderate sensory loss<br>2=severe to total sensory loss (also in comatose patients, tetraplegia)					
9. Best language<br>0=no aphasia, normal<br>1=mild to moderate aphasia<br>2=severe aphasia<br>3=mute, global aphasia (also in comatose patients)					
10. Dysarthria<br>0=normal<br>1=mild to moderate dysarthria<br>2=severe dysarthria (also in comatose patients)					
11. Neglect (bilat. simultaneous stimuli of vision, skin sensation)<br>0=no abnormality<br>1=neglect in one sensory modality<br>2=neglect in both sensory modalities					
**TOTAL NIHSS SCORE**					

NIH Stroke Scale (NIHSS)		Ambulanse	Mottak	2t	Dag1	Utreise
Benytt første respons som mål for funksjon<br>Ikke instruer Pasienten (med mindre det er spesifisert i teksten)<br>Noen punkter scores bare hvis de er til stede (f.eks koordinasjon)<br>Notér hva pasienten gjøre, ikke hva du tror pasienten kan gjøre	Dato					
	Tid					
1a. Bevissthetsnivå<br>0=Våken<br>1=Døsig, reagerer adekvat ved lett stimulering<br>2=Døstig, reagerer først ved kraftig/gjentatt stimulering<br>3=Reagerer ikke, eller bare med ikke-målrettet bevegelse						
1b. Orientering (spør om måned + alder)<br>0=Svarer riktig på to spørsmål<br>1=Svarer riktig på ett spørsmål (eller ved alvorlig dysartri)<br>2=Svarer ikke riktig på noe spørsmål						
1c. Respons på kommando (lukke øyne + knyte hånd)<br>0=Utfører begge kommandoer korrekt<br>1=Utfører en kommando korrekt<br>2=Utfører ingen korrekt						
2. Blikkbevegelse (horisontal bevegelse til begge sider)<br>0=Normal<br>1=Delvis blikkparese (eller ved øyemuskelparese).<br>2=Fiksert blikkdreining til siden eller total blikkparese						
3. Synsfelt (bevegelse fingre/fingertelling laterale synsfelt)<br>0=Normalt<br>1=Delvis hemianopsi<br>2=Total hemianopsi<br>3=Bilateral hemianopsi (blind)						
4. Ansikt (vise tenner, knipe igjen øynene, løfte øyebryn)<br>0=Normal<br>1=Utvisket nasolabialfure, asymmetri ved smil<br>2=Betydelig lammelse i nedre ansiktshalvdel<br>3=Total lammelse i halve ansiktet (eller ved coma)						
5. Kraft i armen (holde armen utstrakt i 45º i 10 sekunder)<br>0=Normal (også ved ikke testbar)<br>1=Drifter til lavere posisjon	Venstre					
2=Noe bevegelse mot tyngdekraften, drifter til sengen<br>3=Kun små muskelbevegelser, faller til sengen<br>4=Ingen bevegelse	Høyre					
6. Kraft I benet (holde benet utstrakt i 30º i 5 sekunder)<br>0=Normal (også ved ikke testbar)<br>1=Drifter til lavere posisjon	Venstre					
2=Noe bevegelse mot tyngdekraften, drifter til sengen<br>3=Ingen bevegelse mot tyngdekraften, faller til sengen<br>4=Ingen bevegelse	Høyre					
7. Koordinasjon / ataksi (finger-nese-prøve/hæl-kne-prøve)<br>0=Normal (også ved ikke testbar eller ved coma)<br>1=Ataksi I arm eller ben<br>2=Ataksi I arm og ben						
8. Hudfølelse (sensibilitet for stikk)<br>0=Normal (også ved ikke testbar)<br>1=Lettere sensibilitetsnedsettelse<br>2=Markert sensibilitetstap (også ved coma, tetraparese)						
9. Språk / afasi (tale, taleforståelse, leseforståelse, benevning)<br>0=Normal<br>1=Moderat afasi, samtale mulig<br>2=Markert afasi, samtale svært vanskelig eller umulig<br>3=Ikke språk (også ved coma)						
10. Tale / dysartri (spontan tale)<br>0=Normal<br>1=Mild - moderate dysartri<br>2=Nær uforståelig tale eller anartri (også ved coma)						
11. Neglect (bilat. Simultan stimuli av syn og hudfølelse)<br>0=Normal (også ved hemianopsi med normal sensibilitet)<br>1=Neglect I en sansemodalitet<br>2=Neglect i begge sansemodaliteter						
**TOTAL NIHSS SCORE**						
