# Supplementary figures and images for: Interrater Agreement on National Institutes of Health Stroke Scale Between Paramedics and Stroke Physicians: Validation Study for the Digital Training Model in the Paramedic Norwegian Acute Stroke Prehospital Project
Source: JMIR Neurotechnol. 2022 Aug 11;1(1):e39444. doi: 10.2196/39444 (PMC12671290; doi:10.2196/39444)

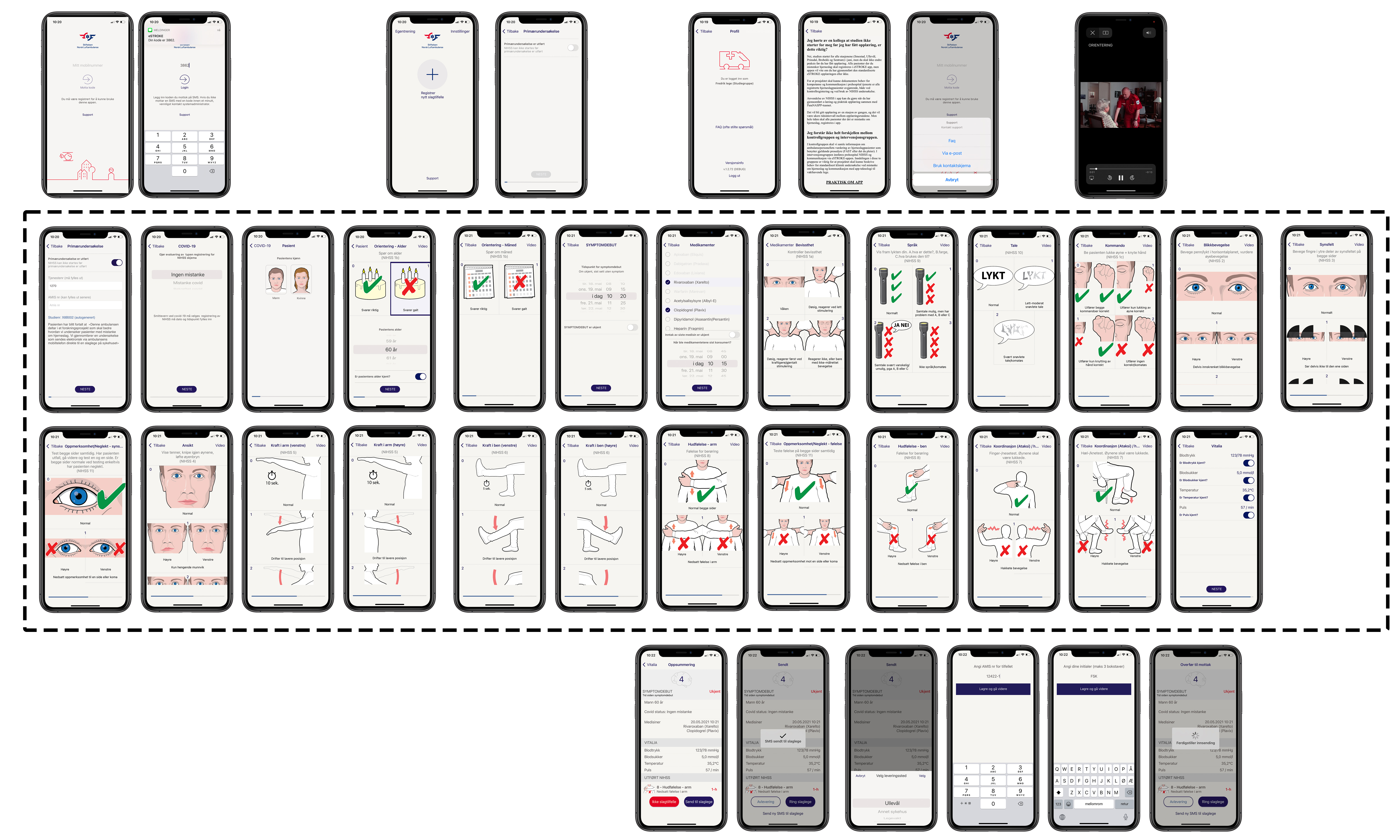

Supplement: Multimedia Appendix 2 [file neuro_v1i1e39444_app2.png]
